# Similar views on rehabilitation following hip arthroscopy among physiotherapists and surgeons in Scandinavia: a specialized care survey

**DOI:** 10.1007/s00167-017-4676-6

**Published:** 2017-08-14

**Authors:** T. Wörner, K. Thorborg, H. Moksnes, F. Eek

**Affiliations:** 1 Department of Health Sciences, Lund University, Box 157, 22100 Lund, Sweden; 2 Sports Orthopaedic Research Center (SORC-C), Department of Orthopaedic Surgery, Copenhagen University Hospital, Amager-Hvidovre, Copenhagen, Denmark; 3 Oslo Sports Trauma Research Center, Norwegian School of Sports Sciences, Oslo, Norway; 4 The Olympic Elite Sports Program (Olympiatoppen), Oslo, Norway

**Keywords:** Hip joint, FAI, Arthroscopy, Rehabilitation, Physiotherapy

## Abstract

**Purpose:**

The rising number of hip arthroscopies (HA) is leading to increasing numbers of patients requiring post-surgical rehabilitation; however, evidence regarding post-operative rehabilitation is currently limited. The purpose of the study was to describe and compare current rehabilitation strategies and views among surgeons and physiotherapists in Scandinavia.

**Methods:**

Scandinavian surgeons and physiotherapists experienced with HA and post-surgical rehabilitation were asked to complete an online survey. Ninety clinicians (28 surgeons, 62 physiotherapists) responded.

**Results:**

Both professions mostly rated physiotherapy as very or extremely important in the rehabilitation process. The majority advocated criteria-based or combined criteria- and time-based progression. Expected rehabilitation timelines were reported with large intra-professional variation but general inter-professional agreement. However, compared with physiotherapists surgeons expected fewer weeks on crutches and faster return to competitive sport. Surgeons more often reported use of evidence-based self-reported outcomes while physiotherapists more often evaluated readiness for return to play.

**Conclusions:**

Among surgeons and physiotherapists, physiotherapy is considered very important following HA. Generally, very similar views were held between professions. Surgeons expected reduced time on crutches and to return to competitive sports than physiotherapists. Surgeons also used evidence-based self-reported outcomes to a higher degree than physiotherapists. Being the first study to provide an overview on currently applied rehabilitation strategies following HA, results of this study may guide much needed, future research on the rehabilitation process following HA.

**Level of evidence:**

IV.

## Introduction

Hip arthroscopy (HA) is used to treat a variety of intra- and extra-articular pathologies [3]. The worldwide number of HAs being performed is increasing [7, 9, 25, 34], with a continued rise in numbers expected [21]. Alongside this rise, increasing numbers of patients are requiring post-surgical rehabilitation.

Current Scandinavian research on HA consists of a limited number of studies evaluating outcomes following surgery [11, 23, 28, 31, 32], but there have been efforts to initiate national HA registries [26, 30]. From an international perspective, there is a paucity of information regarding post-operative rehabilitation despite it being an integral part of the outcome [8, 18]. Only one Scandinavian study, investigating post-surgical outcomes, has reported details regarding post-surgical rehabilitation [12]. Systematic reviews investigating rehabilitation following HA report that the majority of publications are clinical commentaries describing a variety of poorly reported rehabilitation protocols and express the need for further research within this field [8, 18].

Current evidence on rehabilitation following HA is limited to individual expert opinion and experience-based protocols. There is a need to bridge the gap between clinical practice and available evidence and for universal consensus regarding rehabilitation guidelines [8]. The extent to which orthopaedic surgeons performing HA advocate physiotherapist-led rehabilitation, as recommended at the Warwick hip arthroscopy multidisciplinary agreement meeting [17], is currently unknown. Furthermore, insight regarding opinions on post-surgical restrictions and expected timelines for rehabilitation between surgeons and physiotherapists is currently lacking. To address this gap in current knowledge, it is necessary to describe rehabilitation practices following HA. Evaluation of clinicians’ perspectives regarding the rehabilitation process may show where clinicians have similar or opposing views. Observed differences may identify potential targets for future studies investigating specifics of the rehabilitation process.

The aim of this study is to provide an overview of the rehabilitation process following HA in Scandinavia. Current practice and perspectives regarding rehabilitation strategies among surgeons and physiotherapists providing specialized care within this field will be described. Furthermore, potential differences in perspectives on the rehabilitation process between professions will be explored.

## Materials and methods

Scandinavian (Denmark, Norway, and Sweden) surgeons and physiotherapists experienced with HA and post-surgical rehabilitation were invited to participate in a web-based survey. A combination of convenience and snowball sampling was applied. Orthopaedic surgeons were primarily identified through participant lists of Scandinavian HA meetings. The list was complemented by crosschecking participant lists from the national Scandinavian HA meetings. Finally, surgical departments of clinics and hospitals involved in the Scandinavian ACL-registries were contacted. Physiotherapists were primarily invited through national sports medicine organizations via e-mail and social media. As a second step, physiotherapists were identified through referral patterns, reported by surgeons, as well as through clinics and hospitals involved in the ACL-registries with rehabilitation departments. Potential participants received an initial e-mail invitation to participate in the study during May and June 2016. Two reminders were sent 1 and 3 weeks after initial invitation. A total of 90 clinicians (62 physiotherapists, 28 orthopaedic surgeons) responded to the survey. Subject characteristics are summarized in Table 1.

**Table 1:** Subject characteristics

	Physiotherapists (*n* = 62)	Surgeons (*n* = 28)
Country [% (*n*)]		
Denmark	37.1 (23)	42.9 (12)
Norway	6.5 (4)	21.4 (6)
Sweden	56.5 (35)	35.7 (10)
Gender [% (*n*)]		
Females	40.3 (25)	–
Males	59.7 (37)	100 (28)
Working sector [% (*n*)]		
Private sector	58.1 (36)	32.1 (9)
Public sector	25.8 (16)	46.4 (13)
Public and private sector	16.1 (10)	21.4 (6)
Primary care providers [% (*n*)]	49.2 (30)	3.7 (1)
Specialists [% (*n*)]	50.8 (31)	96.3 (26)
Working at clinic providing both, surgery and rehabilitation [% (*n*)]	38.7 (24)	71.4 (20)
Experience with treatment of HA patients in years		
Mean (SD)	5.6 (3.42)	8.4 (6.05)
Median (IQR)	5 (3–8)	6.5 (4–11.75)
HA patients per year		
Mean (SD)	14.5 (22.41)	67.0 (55.03)
Median (IQR)	5 (3–15)	40 (30–108.75)

*n* number of respondents, *HA* hip arthroscopy, *SD* standard deviation, *IQR* interquartile range

### Survey

A web-based survey was developed through a multiple step procedure. The final survey contained 27 questions regarding perceived value of physiotherapy (including different treatment modalities), progression criteria, outcome evaluation strategies, and expected time frames (minimum, maximum, and average expected number of weeks until different rehabilitation endpoints/outcomes). Respondents were asked to complete surveys with regard to a typical HA patient (defined as 25–40 years old with femoroacetabular impingement and chondral/labral injury).

#### Framework for survey content

Due to the absence of national guidelines and evidence-based rehabilitation protocols, the content of the survey was based on best available evidence [8, 18]. With respect to identified gaps in knowledge regarding the rehabilitation process following HA, the survey focused on the following content: (a) timeline of rehabilitation, (b) recommended/applied rehabilitation guidelines including progression criteria (time-based/outcome-based), (c) utilization and choice of clinical outcome measures and (d) specifics of treatment such as treatment frequency and treatment modalities.

#### Question generation

The research group developed questions aiming to cover all contents described above through collaborative discussion. Question and answer options were formulated in English.

#### Face and content validity

The survey was evaluated for face and content validity through discussion with an expert group of clinicians having substantial experience in the performance of arthroscopy and subsequent rehabilitation (one surgeon, two physiotherapists). Results of the expert group meeting were summarized and discussed among the research group before implementation in the survey.

#### Translation

An officially certified translator translated the English version of the survey into Swedish, Danish and Norwegian languages. The Danish, Norwegian and Swedish members of the study group compared translations to originals. Discrepancies between translations and originals were discussed in the group and resolved by consensus.

### Ethics

Participation in the survey was optional, and participants provided informed consent by responding to the survey. As the study did not handle any personal information or sensitive data, include any physical engagement, or in other ways affect the participants, no formal ethical approval was required.

### Statistical analysis

All data were analysed using SPSS Statistics 23 (IBM Software). Descriptive statistics in the form of percentages or mean and standard deviation (for normally distributed numeric data) and/or median and interquartile range (for non-normally distributed numeric- or ordinal-scale data) were applied. Differences between professions were analysed using Chi-square tests for categorical variables and Mann–Whitney *U* tests for numeric data. For group comparisons, five category ordinal scales regarding perceived influence, importance, etc. were dichotomized by collapsing the two highest alternatives (e.g.: extremely/very; always/often) and the three lowest alternatives (e.g.: not at all/never; slightly/sometimes) and subsequently analysed by Chi-square test.

Due to the descriptive nature of the study, no sample size calculation was performed prior to data collection. It was aimed to include as many clinicians as possible from the limited number of individuals comprising the target population.

## Results

**Fig. 1:**
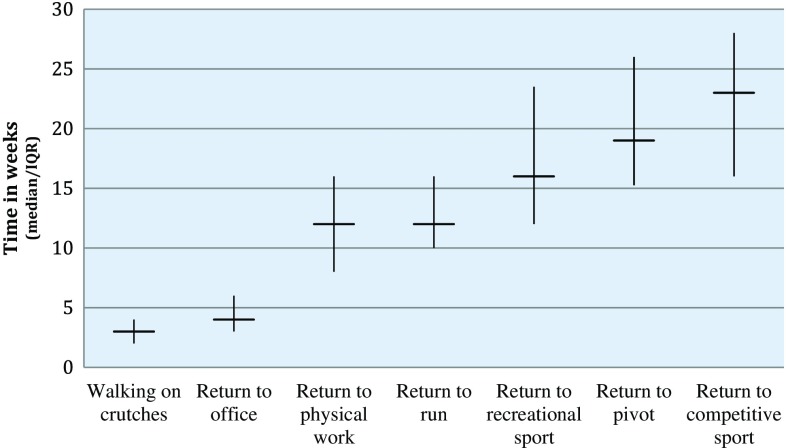
Expected timeline of rehabilitation (professions combined)

**Fig. 2:**
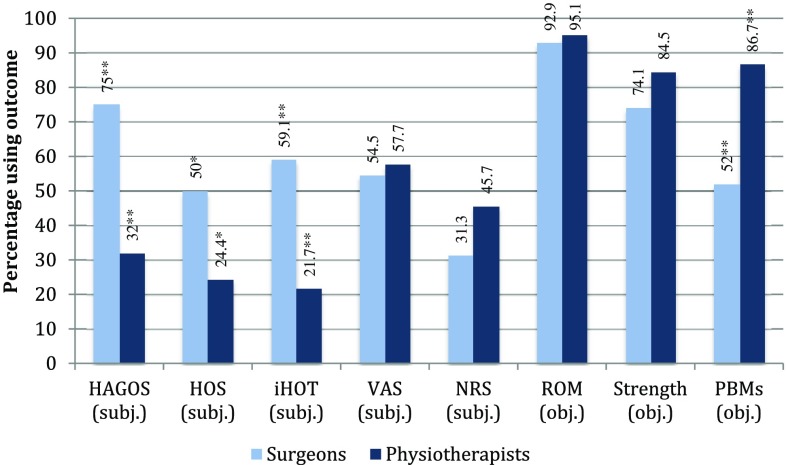
Frequency (%) of used objective and subjective outcomes. *HAGOS* Copenhagen Hip and Groin Outcome Score, *HOS* Hip Outcome Score, *iHOT* International Hip Outcome Tool, *VAS* Visual Analogue Scale, *NRS* Numeric Rating Scale, *ROM* range of motion, *PBMs* performance-based measures, *subj*. subjective, *obj*. objective. *Between group comparison *p* value ≤0.05; **p value ≤0.01

Estimated timeline perspectives regarding rehabilitation milestones, by both surgeons and physiotherapists, are illustrated in Fig. 1. Large within-group variations were observed for timeline perspectives regarding expected milestones. Generally, both professions presented similar views regarding the estimated timeline of rehabilitation. Responses regarding the recommended time on crutches and the expected minimal time to return to competitive sport, however, differed significantly, with surgeons expecting fewer weeks compared with physiotherapists (Table 2). Surgeons more often reported using patient-reported outcomes (PROs) compared with physiotherapists, while physiotherapists more often reported evaluating readiness to return to sport and usage of performance-based measures (PBMs) in the rehabilitation process (Fig. 2 and Table 3).

**Table 2:** Expected timeline of rehabilitation by profession

	Physiotherapists (*n* = 62)			Surgeons (*n* = 28)			Professions combined (*n* = 90)
	AV	Min	Max	AV	Min	Max	AV
Recommended time on crutches in weeks (*n*)	49	60	56	26	26	23	75
Mean (SD)	3.4 (1.45)*	2.3 (1.40)	5.8 (2.68)*	2.6 (1.16)*	1.8 (1.13)	4.5 (2.45)*	3.1 (1.40)
Median (IQR)	4 (2–4)*	2 (1–3)	6 (4–7.5)*	2 (2–3)*	2 (1–2)	4 (3–6)*	3 (2–4)
Return to work in weeks							
Non-physical demanding job (*n*)	44	57	53	25	27	26	69
Mean (SD)	5.4 (3.98)	3.8 (2.78)	9.4 (7.84)	4.7 (2.69)	2.8 (2.13)	8.5 (5.97)	5.1 (3.56)
Median (IQR)	4 (3–7.75)	3 (2–6)	6 (4.5–12)	4 (2.5–6)	2 (1–4)	7 (5.5–12)	4 (3–6)
Physical demanding job (*n*)	43	55	50	25	27	26	68
Mean (SD)	13.0 (5.79)	9.4 (4.08)	19.2 (9.37)	12.6 (4.98)	9.2 (3.97)	19.7 (11.02)	12.8 (5.47)
Median (IQR)	12 (8–16)	8 (6–12)	16 (12–21)	12 (8–15)	8 (6–12)	16 (12–24.5)	12 (8–16)
Recommended time no running in weeks (*n*)	45	58	51	22	25	22	67
Mean (SD)	14.0 (6.18)	10.5 (3.5)	20.8 (11.31)	13.6 (5.91)	9.5 (2.66)	20.6 (11.49)	13.9 (6.05)
Median (IQR)	12 (10–16)	12 (8–12)	16 (12–24)	12 (9.75–16)	10 (8–12)	18 (12–24.5)	12 (10–16)
Recommended time no cut/pivot in weeks (*n*)	43	57	50	21	24	21	64
Mean (SD)	20.8 (9.00)	15.6 (6.04)	30.2 (14.99)	20.0 (7.42)	14.3 (7.18)	30.2 (14.79)	20.5 (8.47)
Median (IQR)	16 (15–28)	12 (12–20)	24 (19–48.5)	20.0 (14–25.5)	12 (10.5–16)	26 (18–45)	19 (15.25–26)
Return to preferred physical activity in weeks							
Recreational level (*n*)	44	58	53	24	24	25	68
Mean (SD)	17.7 (6.91)	13.0 (5.26)	30.2 (14.41)	16.2 (7.02)	12.5 (6.91)	33.3 (20.92)	17.2 (6.93)
Median (IQR)	16 (12.5–23.5)	12.0 (12–16)	25 (20–45)	16 (10.5–23)	12 (8–16)	25 (20–51.5)	16 (12–23.5)
Competitive level (*n*)	41	54	50	24	25	24	65
Mean (SD)	25.1 (11.82)	19.4 (8.75)*	40.3 (14.13)	20.8 (6.38)	15.2 (7.31)*	35.8 (13.13)	23.5 (10.32)
Median (IQR)	24 (16–32)	18 (12–24)*	43 (28–52)	20 (16–24.75)	12 (12–20)*	34 (24–51.5)	23 (16–28)

*n* Number of respondents, *SD* standard deviation, *IQR* interquartile range, *AV* average, *Min* minimum, *Max* maximum

* Between group comparison *p* < 0.05

**Table 3:** Rehabilitation structure and content

Profession (*n*)	Physiotherapists (62)	Surgeons (28)	*p* value*
Patients received by referral [% (*n*)]	48.4 (30/62)	–	–
Patients referred to physiotherapist [% (*n*)]	–	96.4 (27/28)	–
Rated importance of physiotherapy^a^ [% (*n*)]	91.9 (57/62)	82.1 (23/28)	N. S
Number of physiotherapy meetings per month			
Median (IQR)	4 (2–6)	–	–
Number of surgical follow-ups			
Median (IQR)	–	2 (2–2)	–
Specific protocol followed/recommended [% (*n*)]	61.3 (38/62)	72 (18/25)	N. S
Protocol criteria-based/criteria- and time-based [% (*n*)]	86.7 (52/60)	77.8 (21/27)	N. S
Rated high importance of^a^			
Exercise therapy [% (*n*)]	98.4 (60/61)	85.2 (23/27)	0.029
Manual therapy [% (*n*)]	18 (11/61)	25 (7/28)	N. S
Electro-physical modalities [% (*n*)]	1.7 (1/60)	0 (0/28)	N. S
Applied evaluation of treatment by^b^			
Subjective outcomes [% (*n*)]	91.4 (53/58)	100 (26/26)	N. S
Objective outcomes [% (*n*)]	91.3 (52/56)	96.3 (26/27)	N. S
Evaluation of readiness to return to sport (RTS)^c^ [% (*n*)]	74.2 (46/62)	50 (14/28)	0.024
Influence on RTS decision^d^			
Patient [% (*n*)]	80.3 (49/61)	75 (21/28)	N. S
Physiotherapist [% (*n*)]	60.7 (37/61)	46.4 (13/28)	N. S
Surgeon [% (*n*)]	48.4 (29/60)	39.3 (11/28)	N. S

*n* Number of respondents

* Between group comparison, Chi square test

^a^ Respondents rating respective modality as either “extremely important” or “very important”

^b^ Respondents reporting to “sometimes”/“always” evaluate treatment by subjective/objective outcomes

^c^ Respondents reporting to evaluate readiness to return to sport

^d^ Respondents rating the influence of respective roles in the return to sport decision process as “extremely influential” or “very influential”

**Fig. 3:**
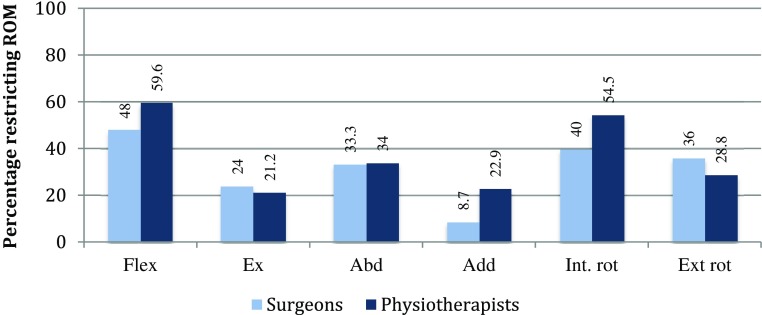
Frequency (%) of recommended post-surgical ROM-restrictions. *ROM* range of motion, *Flex* flexion, *Ex* extension, *Abd* abduction, *Add* adduction, *Int. rot* internal rotation, *Ext rot* external rotation

**Fig. 4:**
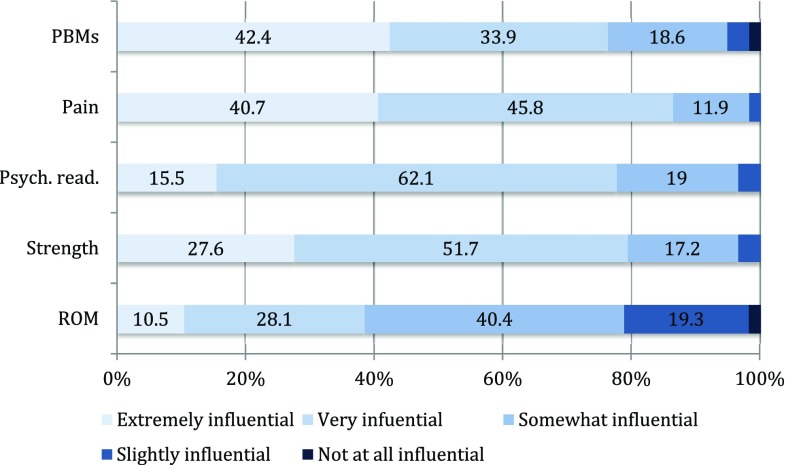
Influence of different outcomes on RTS decision. *RTS* return to sport, *PBMs* performance-based measures, *Psych. read* psychological readiness, *ROM* range of motion, percentages (%) are displayed when exceeding 10% of the study sample

Recommendations of post-surgical range of motion (ROM) restrictions are summarized in Fig. 3. Participants’ ratings of influence of clinical outcomes on the return to sport (RTS) decision are illustrated in Fig. 4. Physiotherapists more often than surgeon-rated strength (physiotherapists: 88.9%, surgeons: 46.3%; *p* = 0.003) and performance-based measures (physiotherapists: 84.8%, surgeons: 46.2%; *p* = 0.008) to be influential in the RTS decision.

## Discussion

This is the first study to investigate current clinical practice in rehabilitation following HA, as implemented by surgeons and physiotherapists. Previous studies have only included post-surgical management from surgeons’ perspectives [14, 19].

Physiotherapy was rated to be very important in rehabilitation following HA by both professions. These results are in line with the Warwick agreement recommending physiotherapist-led rehabilitation as the cornerstone of rehabilitation [17]. In general, both professions presented similar views on the rehabilitation process. More than 75% of respondents recommend either criteria-based or combined criteria- and time-based rehabilitation progression. Published rehabilitation protocols typically describe rehabilitation progression based on functional criteria and estimated tissue healing times [13, 15, 35, 38, 39]; however, there is no current evidence favouring one specific approach. Rehabilitation protocols are generally poorly reported and demonstrate large variability [8, 18]. Until results of comparative trials are published [4, 37], clinical opinions will likely vary. Therefore, uncertainty in best practice may explain the general variability regarding the expected timeline of rehabilitation observed in our study.

More optimistic views regarding minimal expected time to return to competitive sports following HA were expressed by the surgeons in our study than by the physiotherapists. This might be due to surgeons basing recommendations on biological healing times versus physiotherapists basing recommendations on clinically observable progression criteria such as normalization of pain-free gait patterns [18]. Although time to RTS is rarely reported [33] and varies greatly [5, 6, 27, 29], a recent meta-analysis reported that patient-reported improvements in sport function occur between 6 months and 1 year post-surgery [22]. However, similar to our results, surgeons from high volume HA centres recommended 12–20 weeks for athletes to return to competitive sports [14]. An objective evaluation of health status is needed to guide the athlete towards an informed RTS decision [10]. According to our results, physiotherapists more frequently evaluate RTS and rate objective measures such as PBMs and strength as very important in the RTS decision, compared with surgeons. Such objective clinical outcomes are more easily collected during frequent clinical sessions, which may be a possible explanation for the difference in use we found. This difference in direct involvement in the RTS decision could potentially also explain the difference in minimal expected time to RTS.

Generally, a combination of subjective and objective outcomes is recommended for evaluation of results of arthroscopy and following rehabilitation [20]. Surgeons more frequently reported use of PROs such as HAGOS, iHOT and HOS, which are recommended for evaluation of treatment efficacy of HA and following rehabilitation [18, 36]. Considering the fact that physiotherapists meet patients approximately four times a month, we find it surprising that not more of them use evidence-based PROs to monitor rehabilitation progression and evaluate treatment efficacy. The differing clinical working routines between professions may explain why surgeons more often use PROs, while physiotherapists more often use PBMs, in the evaluation of post-surgical outcomes. About 40% of physiotherapists and 71% of all surgeons in our study work at clinics providing both surgery and rehabilitation, and it is possible that PROs and PBMs collected by either profession, or via routine clinical follow-up, are shared between professions.

Despite being frequently advocated in current literature [15, 24, 35, 38, 39], 80% of clinicians in our study rate passive modalities such as manual therapy less important than exercise therapy, which was rated very important by almost all responding clinicians. Early restoration of motion including pain-free joint ROM is generally encouraged [18] and more than half of surgeons in our study do not recommend any restrictions in ROM following HA. There is conflicting evidence regarding improvements of ROM following HA [16] and participants in our study rated ROM to be the least influential factor in the RTS decision. The primary symptom of FAI-syndrome is pain [17], and one of the main goals of HA is to relieve pain [2]. Therefore, it is not surprising that the participants rated pain as the most influential measure in the RTS decision. Almost 80% of responding clinicians rated psychological readiness to be very influential in the RTS decision. Psychological readiness is considered an important aspect in this decision [1] but has, to our best knowledge, not been investigated in patients following HA.

A number of limitations in the current study exist. Surgeons were invited to participate by identification through participation lists of national and Scandinavian HA meetings, which led to confidence in having approached the majority of them. However, it is possible that surgeons with interest in rehabilitation were more likely to take part in the survey. This may have led to an overestimation of positive attitude towards physiotherapy. Physiotherapists were approached via sports medicine organizations using e-mail and through social media. By identifying surgical centres specialized in arthroscopy through the Scandinavian ACL-registries, contacting their respective rehabilitation departments, and through our analysis of surgeons’ referral patterns, we aimed to reduce selection bias.

Considering the primarily descriptive nature of the study and the limited size of the total target population, no sample size calculation was performed prior to recruitment. Due to the inherent small sample size associated with the specialist clinician population investigated, a risk of type 2 error in the comparison of professions exists.

Little is known about the rehabilitation process following hip arthroscopy, and more research on the topic is warranted [8, 18]. This study provides a reflection of current usual care in the rehabilitation following HA for patients in Scandinavia. By investigating care practices and opinions, results of this study may instigate first steps towards establishing clinical consensus for rehabilitation following hip arthroscopy and highlight areas for future research.

## Conclusions

Physiotherapists and surgeons presented very similar views on the rehabilitation process. Physiotherapy is considered very important following HA by both professions. The majority of respondents advocate either criteria-based or combined criteria- and time-based rehabilitation progression. Surgeons expected shorter time on crutches and to return to competitive sports than physiotherapists. Surgeons also used evidence-based self-reported outcomes to a greater extent than physiotherapists.
